# Primary Thyroid Lymphoma: An Analysis of the National Cancer Database

**DOI:** 10.7759/cureus.4088

**Published:** 2019-02-18

**Authors:** Victoria Vardell Noble, Daniel A Ermann, Emily K Griffin, Peter T Silberstein

**Affiliations:** 1 Internal Medicine, Creighton University School of Medicine, Omaha, USA; 2 Oncology, Creighton University School of Medicine, Omaha, USA

**Keywords:** extra nodal lymphoma, ncdb, national cancer database, thyroid malignancy, chop, dlbcl, nhl, mantle cell lymphoma, thyroid lymphoma, non-hodgkin lymphoma

## Abstract

Introduction

Primary thyroid lymphoma (PTL) is a rare malignancy, representing only 1% to 5% of thyroid malignancies and 2.5% to 7% of all extranodal lymphomas. Most cases of PTL are of B-cell origin, and 98% of all PTL cases are non-Hodgkin’s lymphoma. Case series and case reports represent the majority of the available studies on PTL, with a paucity of large retrospective population studies available for this disease. This is the first National Cancer Database (NCDB) study completed on PTL and the only large retrospective study to examine the use of chemotherapy and immunotherapy in the treatment of this specific population.

Methods

The NCDB for non-Hodgkin's lymphoma was utilized to identify 3,466 patients diagnosed with PTL between 2004 and 2015. The database was used to examine demographic information including age, race, gender, histology, stage, and treatment modality. Bivariate Kaplan-Meier analysis with log-rank tests was used to analyze overall survival. Multivariate analysis was performed with Cox proportional hazards regression models to obtain hazard ratios to assess the association of patient characteristics and treatment methods with survival.

Results

The median all-cause survival for PTL was 11.6 years (95% confidence interval [CI]: 11.1 to 12.1 years). The majority of PTL patients were female (68%) and white (93%), with a mean age of 65.8 years. Histologically, 59.5% of cases were diffuse large B-cell lymphoma (DLBCL), 18.3% marginal zone lymphoma, 8% follicular lymphoma, and 1.9% Burkitt lymphoma. Regarding treatment, 40.6% received beam radiation, and 54% underwent surgical resection. Single-agent chemotherapy was used in only 3.5% of patients, where 60.7% received multiagent chemotherapy. Additionally, immunotherapy was used in 16.2% of patients.

There was a significantly increased risk of mortality associated with increasing age, DLBCL histology, and higher disease stage. Multivariate analysis of treatment methods revealed that lobectomy (hazard ratio [HR]: 0.58, 95% CI: 0.47-0.73) and total or subtotal thyroidectomy (HR: 0.58, 95% CI: 0.47-0.71) had significantly improved survival rates over no surgical management (*p *< 0.001). Beam radiation (HR 0.67, 95% CI: 0.58-0.79) had a significant survival benefit over treatment regimens that did not include radiation therapy (*p *< 0.001). Multiagent (HR: 0.40, 95% CI: 0.33-0.49) and single-agent chemotherapy (HR: 0.43, 95% CI: 0.30-0.63) had significant improvement over treatment regimens that did not include chemotherapy (*p *< 0.001). Immunotherapy had a survival benefit (HR 0.87) although this was not found to be statistically significant (95% CI: 0.68-1.11). Other factors associated with decreased risk of mortality include treatment at academic medical centers (HR: 0.846) and integrated cancer centers (HR: 0.76) as compared to community centers (*p *< 0.05).

Conclusion

This is the largest study to date of PTL and the first to analyze the NCDB database. Patient characteristics, treatment modalities, and overall survival in PTL were examined to further characterize this rare disease. Beam radiation, chemotherapy, and surgical resection all reveal significant survival benefit, with multiagent chemotherapy having the greatest advantage.

## Introduction

Primary thyroid lymphoma (PTL) is a rare lymphoma that develops in the thyroid gland, representing only 1% to 5% of thyroid malignancies and 2.5% to 7% of all extra-nodal lymphomas [1-5]. The largest study to date describing this disease by Graff-Baker, et al. included 1408 cases in the Surveillance, Epidemiology, and End Results (SEER) database [3]. Graff-Baker, et al. found that 98% of all PTL is non-Hodgkin’s lymphoma [3]. This study observed that 68% of cases of PTL are histologically diffuse large B-cell lymphoma (DLBCL); with other histological subtypes including follicular lymphoma (10%), marginal zone or mucosal-associated lymphoid tissue (MALT) lymphoma (10%), and small lymphocytic lymphoma (3%) [3]. DLBCL represents the highest grade of PTL with the most aggressive course [2-3,6]. MALT lymphoma is considered indolent and low grade, representing a benign subtype of PTL [2-4.6]. DLBCL can develop from MALT lymphoma, and rarely the two can be found together in a mixed subtype [2,6-8].

Graff-Baker, et al. found a median survival of 9.3 years, with the majority of patients being female (75%) and white (93%) with a mean age of 66 years [3]. Prior studies have found that poor prognostic factors include age greater than 80 years, DLBCL histology, and advanced stage [3,6]. Variables associated with improved prognosis include treatment with either radiation, surgery, or multiagent chemotherapy [3,6].

PTL generally presents with a rapidly enlarging anterior neck mass that may cause obstructive symptoms and be associated with cervical lymphadenopathy [2]. Up to 10% of patients present with B type symptoms such as weight loss, night sweats, and fever [2]. PTL is generally diagnosed by either fine needle aspiration (FNA) or open biopsy, as PTL is difficult to differentiate from other malignancies or benign processes by ultrasound alone [4-6,8].

There is a paucity of large retrospective population studies available for this disease. As case series and case reports represent the majority of the available studies on PTL, optimal treatment methods have not been well established. PTL is generally treated with a combination of chemotherapy and loco-regional radiotherapy, with surgery generally reserved for cases where major debulking is required [2,4,7,9]. Chemotherapy generally involves the CHOP regimen of cyclophosphamide, doxorubicin, vincristine, and prednisone, though other combinations may be used [2]. Rituximab is usually added to this combination, with CHOP plus rituximab showing promising results in the management of PTL [2]. Radiotherapy of 30-60 Gray (Gy) is the standard adjuvant therapy when beam radiation is used [9].

This study includes the largest group of PTL patients studied to date, and the first study using the National Cancer Database (NCDB) to analyze the demographics and prognosis of PTL. This is also the first retrospective population study to examine the use of chemotherapy and immunotherapy in the prognosis of PTL.

## Materials and methods

The NCDB is the largest clinical registry in the world, representing over 70% of all new invasive cancer diagnosis in the United States every year. It is a project of the American Academy of Surgeons (ACoS), the Commission on Cancer (CoC), and the American Cancer Society and has been collecting data on newly diagnosed cancers since 1985 [10]. 

The NCDB participant user files for extra-nodal and nodal non-Hodgkin’s lymphoma were combined and used to identify patients diagnosed with PTL between 2004 and 2015. Primary thyroid site was determined using the International Classification of Diseases for Oncology, 3rd Edition (ICD-O-3) topographical code, C73.9. 

Only non-Hodgkin’s lymphoma was included, and histological subtypes were examined by ICD-O-3 morphological specific codes. Histologies present include lymphoma, not otherwise specified (NOS) (ICD-O-3 9590-9591), composite-site Hodgkin’s lymphoma (ICD-O-3 9596), small lymphocytic B cell (ICD-O-3 9670-9671), mantle cell lymphoma (ICD-O-3 9673), mixed diffuse B cell (ICD-O-3 9675), thymic large B cell lymphoma (ICD-O-3 9679), DLBCL (ICD-O-3 9680-9684), Burkitt’s lymphoma (ICD-O-3 9687), follicular lymphoma (ICD-O-3 9690-9698), marginal zone, or MALT, lymphoma (ICD-O-3 9699), and T-cell lymphoma (ICD-O-3 9702-9714).

The examined demographic characteristics included sex, insurance status, facility type (community, comprehensive community, academic, and integrated centers), location, Charleson/Deyo comorbidity index score, and HIV status. The NCDB describes community centers as facilities treating between 100 and 500 newly diagnosed cancer cases each year, where comprehensive centers treat >500 new cases per year. Academic centers participate in postgraduate medical education and participate in cancer-related research. Race was separated into black, white, and all other categories, with Hispanic or non-Hispanic identified separately. Year of diagnosis was examined for all years included in the database, from 2004 to 2015, and separated into four-year periods. In examining tumor characteristics and presentation, the stage was compared using the Ann Arbor classification as detailed in the American Joint Committee of Cancer (AJCC) Staging manual, 7th edition [11]. The AJCC Ann Arbor classification defines that stage I PTL is confined to the thyroid, stage II involves lymph nodes on the same side of the diaphragm, stage III involves lymph nodes on the opposite side of the diaphragm, and stage IV has distant metastasis present at diagnosis [11]. Histologic subtypes found in greater than 1% of PTL patients were included in analysis: DLBCL, Burkitt’s lymphoma, follicular lymphoma, marginal and MALT lymphoma, and lymphoma NOS. The presence or absence of systemic “B symptoms” such as night sweats, fever, or weight loss, was also included. 

Management of PTL was examined by the type and sequence. Characteristics compared include surgical treatment methods, where site-specific surgical codes were used to identify methods of surgical treatment (no surgical management, partial lobectomy or local excision, lobectomy, and subtotal or total thyroidectomy). The use of beam radiation, radioisotopes, or no radiation and the sequence of radiation and surgery, either before or after surgery, were included as separate groups. The use of single- or multi-agent chemotherapy, or no chemotherapy, as well as the administration of immunotherapy, was included. The sequence of chemotherapy and surgery, before or after surgery, was also examined. 

Cox regression analysis with 95% confidence interval (CI) was used for bivariate analysis of hazard ratios (HR) for evaluating the risk of mortality for demographic, histologic, pathologic, and treatment characteristics. This included evaluation of all previously listed demographic, pathologic, and clinical characteristics, with the exception of human immunodeficiency virus (HIV) status, which was excluded due to the limited data and poor significance. Multivariate analysis of select characteristics was performed using Cox regression analysis with 95% CI. Variables included sex, age, race (white, black, other), insurance status, facility type, Charleson/Deyo comorbidity score, stage, and histology. Clinical characteristics included use and extent of surgery, radiation (none, beam, radioisotope), chemotherapy (none, multiagent, single agent) and the administration of immunotherapy. The presence of systemic symptoms, facility location, and combination therapy and sequence was excluded due to small sample size and low power of results. Results are reported as HRs for mortality with 95% CI. Two-sided tests of significance were performed and evaluated for a significance of *p* > 0.05. 

Bivariate Kaplan-Meier analysis was used to evaluate survival from all-cause mortality. Overall survival and characteristic specific survival were also examined by stage, histology, and by use and type of surgical, radiation, and chemotherapeutic management. Significance was evaluated with log-rank tests to evaluate the significance of *p* >0.05. Survival tables for cumulative survival from all-cause mortality were used for five- and 10-year overall survival and to compare five- and10-year survival by stage and histology. Wilcoxin (Gehan) statistics were used to determine significance at *p* <0.05.

All analysis was completed using the Statistical Package for Social Sciences (SPSS) software version 25. Institutional Review Board (IRB) exemption was obtained due to the use of a de-identified public database.

## Results

Patient, tumor, and treatment characteristics for 3,466 patients with PTL are listed in Table 1. Most patients were female (68.6%) with a normally distributed average age at diagnosis of 65.8 years (+/-14 years). The majority were white (93%), with only 2.2% identifying as black, while 94.5% of all patients identified as non-Hispanic. The majority of patients were insured by Medicare (51.4%), with private insurance representing the next largest group (40.8%). Medicaid (4.2%) and uninsured (2.5%) represented a minority of patients. Most patients received treatment at comprehensive community centers (43.9%) followed by academic centers (36.6%), with only a small group (9%) receiving treatment at community hospitals. The location of these facilities revealed a majority receiving treatment in the South Atlantic region (states of DC, DE, FL, GA, MD, NC, SC, VA, WV) and the East North Central region (states of IL, IN, MI, OH, WI), at 20.2% and 20%, respectively. The large majority of patients with known HIV status were HIV negative (99%).

**Table 1:** Demographics, pathologic characteristics and treatment methods for primary thyroid lymphoma, from the NCDB, 2004-2015 SD, standard deviation; CCC, Comprehensive Community Center; NCDB, National Cancer Database

Patient Demographics		N	%		Characteristics		N	%
Sex (n = 3466)					Stage (n = 3156)			
	Male	1080	31.2			I	1728	54.8
	Female	2386	68.8			II	973	30.8
Age (n = 3466)						III	149	4.7
	Mean age	65.8 (+/- 14.1 SD)				IV	306	9.7
					Histology (n = 3382)			
	<21	10	0.3			DLBCL	2070	61.2
	21-30	33	1			Burkitt	66	2
	31-40	106	3.1			Follicular	274	8.1
	41-50	350	10.1			Marginal	634	18.7
	51-60	725	20.9			Lymphoma, NOS	338	10
	61-70	848	24.5					
	71-80	839	24.2		Systemic 'B' Symptoms at Diagnosis (n = 2999)			
	>80	555	16			Absent	2644	88.2
Race (n = 3466)						Present	355	11.8
	White	3223	93					
	Black	77	2.2					
	Other	166	4.8		Surgical Treatment (n = 3423)			
Hispanic (n = 3263)						None	1585	46.3
	Non-Hispanic	3085	94.5			Partial lobectomy/ local excision	242	7.1
	Hispanic	178	5.5			Lobectomy	618	18.1
Year of Diagnosis (n = 3466)						Total/Subtotal Thyroidectomy	978	28.6
	2004-2007	1157	33.4		Radiation Therapy (n = 3421)			
	2008-2011	1193	34.4			None	1997	58.4
	2012-2015	1116	32.2			Beam Radiation	1406	41.1
Insurance Status (n = 3417)						Radioisotopes	18	0.5
	Not insured	85	2.5		Surgery Radiation Sequence (n = 3418)			
	Private	1395	40.8			No Radiation &/or Surgery	2647	77.4
	Medicaid	142	4.2			Radiation before Surgery	7	0.2
	Medicare	1755	51.4			Radiaiton after Surgery	764	22.4
	Other Government	40	1.2		Chemotherapy (n=3263)			
Facility Type (n=3338)						None	1036	31.7
	Community	299	9			Single-agent Chemotherapy	122	3.7
	CCC	1456	43.9			Multiagent Chemotherapy	2105	64.5
	Academic	1222	36.6		Immunotherapy (n = 3419)			
	Integrated	361	10.8			None	2865	83.8
Facility Location (n = 3338)						Administered	554	16.2
	New England	176	5.3		Surgery Chemotherapy Sequence (n = 2557)			
	Middle Atlantic	501	15			No Chemotherapy &/or Surgery	1682	65.8
	South Atlantic	668	20			Chemotherapy before Surgery	13	0.5
	East North Central	675	20.2			Chemotherapy after Surgery	862	33.7
	East South Central	297	8.9					
	West North Central	297	8.9					
	West South Central	260	7.8					
	Mountain	131	3.9					
	Pacific	333	10					
Charleson Deyo Score (n = 3466)								
	0	2695	77.8					
	1	591	17.1					
	2	141	4.1					
	3	39	1.1					
HIV Status (n = 1968)								
	HIV Negative	1949	99					
	HIV Positive	19	1					

The majority, 54.8%, of tumors were AJCC 6th edition Ann Arbor Stage I at diagnosis, followed by 30.8% diagnosed at Stage II. The minority of patients were diagnosed at Stage III (4.7%) and Stage IV (9.7%). 

The majority (99.1%) of PTL originates from B-cell precursors, with only 0.9% of T-cell origin. Histologically most tumors are DLBCL (61.2%), followed by marginal zone MALT lymphoma (18.7%), follicular lymphoma (8.1), and Burkitt’s lymphoma (2%), with 10.0% categorized as lymphoma NOS. 

In patients where symptoms at diagnosis were reported, 11.8% reported systemic “B symptoms”.

Approximately half of the patients (53.7%) received surgery in the management of PTL. Total or subtotal thyroidectomy was performed in 28.6% of patients, with lobectomy in 18.1%, and partial lobectomy or local excision in 7.1%. Approximately 42% of patients received radiation therapy, predominantly with beam radiation (41.1% of all patients), and 22.4% received adjuvant radiation therapy following surgical resection. 

Chemotherapy represents the most common treatment, used in 68.3% of all patients. Multi-agent therapy was used in 64.5% of all patients, and only a small minority (3.7%) received a single-agent treatment. Immunotherapy was reportedly used in 16.2% of patients. Adjuvant chemotherapy following surgery was used in the management of 33.7% of patients. 

Bivariate analysis

Demographic, tumor, and management characteristics were analyzed for their effects on all-cause mortality (Table 2). Demographics associated with significant (*p *< 0.05) increased risk of mortality on bivariate analysis included female gender (HR: 1.26), increasing age (HR: 19.5 in those >80), Medicare insurance (HR: 3.0), and Charleson-Deyo scores greater than zero (HR: 1.68, 2.65, and 2.64 for scores of one, two, and three, respectively). Demographic factors associated with improved survival included identification as Hispanic (HR: 0.63) and treatment at facilities other than community centers (HR: 0.76 for comprehensive community centers, HR: 0.64 for academic centers, and HR: 0.66 for integrated centers). 

**Table 2:** Hazard ratios for all-cause mortality from bivariate analysis of demographic, pathologic, and clinical characteristics of primary thyroid lymphoma patients from the NCDB, 2004-2015 *Reference; NS, not statistically significant (*p* > 0.05); CCC, Comprehensive Community Center; NCDB, National Cancer Database

Patient Demographics+B58:J104		Hazard Ratio (HR)	p value		Characteristics		Hazard Ratio (HR)	p value
Sex (n=3466)					Stage (n=3156)			
	Male	1	*			I	1	*
	Female	1.26	<0.01			II	1.12	NS
Age (n=3466)						III	1.32	NS
	<51	1	*			IV	1.74	<0.001
	51-60	1.76	<0.01		Histology (n= 3382)			
	61-70	3.55	<0.001			DLBCL	1	*
	71-80	7.94	<0.001			Burkitt	0.65	NS
	>80	19.50	<0.001			Follicular	0.51	<0.001
Race (n=3466)						Marginal	0.55	<0.001
	White	1	*			Lymphoma, NOS	1.08	NS
	Black	1.03	NS					
	Other	0.71	NS		Systemic B symptoms present at Diagnosis (n=2999)			
Hispanic (n=3263)						Absent	1	*
	Non-Hispanic	1	*			Present	1.53	<0.001
	Hispanic	0.63	<0.05					
Year of Diagnosis					Clinical			
	2004 - 2007	1	*		Surgical Treatment (n=3423)			
	2008 - 2011	0.99	NS			None	1	*
	2012 - 2015	0.82	NS			Partial lobectomy/ local excision	0.85	NS
Insurance Status (n=3417)						Lobectomy	0.52	<0.001
	Not insured	1	*			Total/ Subtotal Thyroidectomy	0.49	<0.001
	Private	0.61	NS		Radiation Therapy (n=3421)			
	Medicaid	1.01	NS			None	1	*
	Medicare	3.00	<0.001			Beam Radiation	0.61	<0.001
	Other Government	2.29	<0.05			Radioisotopes	1.05	NS
Facility Type (n=3338)					Surgery Radiation Sequence (n=3418)			
	Community	1	*			No Radiation &/or Surgery	1	*
	CCC	0.76	<0.05			Radiation before Surgery	1.47	NS
	Academic	0.64	<0.001			Radiaiton after Surgery	0.48	<0.001
	Integrated	0.66	<0.01		Chemotherapy (n=3263)			
Facility Location (n=3338)						None	1	*
	New England	1	*			Single Agent Chemotherapy	0.98	NS
	Middle Atlantic	1.14	NS			Multiagent Chemotherapy	0.71	<0.001
	South Atlantic	1.26	NS		Immunotherapy (n=3419)			
	East North Central	1.13	NS			None	1	*
	East South Central	1.32	NS			Administered	0.89	NS
	West North Central	1.01	NS		Surgery Chemotherapy Sequence (n=2557)			
	West South Central	1.12	NS			No Chemotherapy &/or Surgery	1	*
	Mountain	1.20	NS			Chemotherapy before Surgery	0.21	NS
	Pacific	0.89	NS			Chemotherapy after Surgery	0.56	<0.001
Charleson Deyo Score (n=3466)								
	0	1	*					
	1	1.68	<0.001					
	2	2.65	<0.001					
	3	2.64	<0.001					

Disease characteristics associated with increased risk of mortality on bivariate analysis include higher stage (HR of 1.74 for stage IV) and the presence of systemic ‘B symptoms’ on diagnosis (HR: 1.53). Histology other than DLBCL was associated with decreased risk of mortality, with follicular lymphoma (HR: 0.51) and marginal lymphoma (HR: 0.55) having the lowest risk.

Bivariate analysis of management options revealed improved survival with all types of surgery over no surgery, with significantly improved survival for lobectomy (HR: 0.52) and total or subtotal thyroidectomy (HR: 0.49). Beam radiation had a significant survival benefit (HR: 0.61), as did adjuvant radiation following surgery (HR: 0.48). Chemotherapy with multi-agent therapy had a benefit over no chemotherapy (HR: 0.71), as did chemotherapy following surgery (HR: 0.56). 

Multivariate analysis

Multivariate analysis of patient and tumor characteristics revealed that increasing age, Charleson/Deyo score, and stage remained significant predictors of mortality (Table 3). Treatment at academic and integrated centers (HR: 0.69, 95% CI: 0.53-0.90, and HR: 0.68, 95% CI: 0.49-0.94, respectively) and histology other than DLBCL (follicular HR: 0.51, 95% CI: 0.37-0.71 and marginal lymphoma HR: 0.45, 95% CI: 0.34-0.58) remained significant predictors of improved survival.

**Table 3:** Hazard ratios for all-cause mortality on multivariate analysis of demographic, pathologic, and clinical characteristics for primary thyroid lymphoma, from the NCDB, 2004-2015 (N = 2470) * Reference; NS, Not statistically significant (*p* > 0.05); CCC, Comprehensive Community Center; NCDB, National Cancer Database

Patient Demographics		Hazard Ratio (HR)	95% CI	p value		Characteristics		Hazard Ratio (HR)	95% CI	p value
Sex (n = 3466)						Stage (n = 3156)				
	Male	1		*			I	1		*
	Female	1.02	(0.86-1.21)	NS			II	1.16	(0.98 - 1.38)	NS
Age (n = 3466)							III	1.66	(1.18 - 2.34)	<0.01
	<51	1		*			IV	1.47	(1.16 - 1.86)	<0.01
	51-60	1.64	(0.98-2.76)	NS		Histology (n = 3382)				
	61-70	2.35	(1.42-3.88)	<0.01			DLBCL	1		*
	71-80	4.19	(2.51-6.98)	<0.001			Burkitt	1.11	(0.57-2.18)	NS
	>80	8.94	(5.34-14.99)	<0.001			Follicular	0.51	(0.37-0.71)	<0.001
Race (n=3466)							Marginal	0.45	(0.34-0.58)	<0.001
	White	1		*			Lymphoma, NOS	0.84	(0.66 - 1.08)	NS
	Black	1.46	(0.89-2.39)	NS						
	Other	0.67	(0.43-1.06)	NS						
Insurance Status (n=3417)						Surgical Treatment (n = 3423)				
	Not insured	1		*			None	1		*
	Private	0.46	(0.26-0.82)	<0.01			Partial lobectomy/ local excision	0.93	(0.71-1.21)	NS
	Medicaid	0.68	(0.32-1.46)	NS			Lobectomy	0.58	(0.47-0.73)	<0.001
	Medicare	0.81	(0.45-1.44)	NS			Total/ Subtotal Thyroidectomy	0.58	(0.47 - 0.71)	<0.001
	Other Government	1.71	(0.74-3.93)	NS		Radiation Therapy (n = 3421)				
Facility Type (n = 3338)							None	1		*
	Community	1		*			Beam Radiation	0.67	(0.58-0.79)	<0.001
	CCC	0.82	(0.64-1.06)	NS			Radioisotopes	0.75	(0.23-2.38)	NS
	Academic	0.69	(0.53-0.90)	<0.01		Chemotherapy (n = 3263)				
	Integrated	0.68	(0.49-0.94)	<0.05			None	1		*
Charleson Deyo Score (n=3466)							Single-agent Chemotherapy	0.43	(0.30 - 0.63)	<0.001
	0	1		*			Multiagent Chemotherapy	0.40	(0.33 - 0.49)	<0.001
	1	1.56	(1.30-1.87)	<0.001		Immunotherapy (n = 3419)				
	2	1.80	(1.34-2.42)	<0.001			None	1		*
	3	2.34	(1.33-4.09)	<0.01			Administered	0.87	(0.68-1.11)	NS

Multivariate analysis of treatment characteristics revealed that lobectomy (HR: 0.58, 95% CI: 0.47-0.73) and total or subtotal thyroidectomy (HR: 0.58, 95% CI: 0.47-0.71) had significantly (*p *< 0.001) improved survival over no surgical management. Beam radiation (HR: 0.67, 95% CI: 0.58-0.79) had significant (*p *< 0.001) survival benefit over no radiation therapy, and multiagent (HR: 0.40, 95% CI: 0.33-0.49) and single-agent chemotherapy (HR: 0.43, 95% CI: 0.30-0.63) had significant (*p *< 0.001) improvement over no chemotherapy. Immunotherapy had a survival benefit with HR 0.87, although this was not statistically significant with a 95% CI of 0.68-1.11. 

Survival

**Figure 1:**
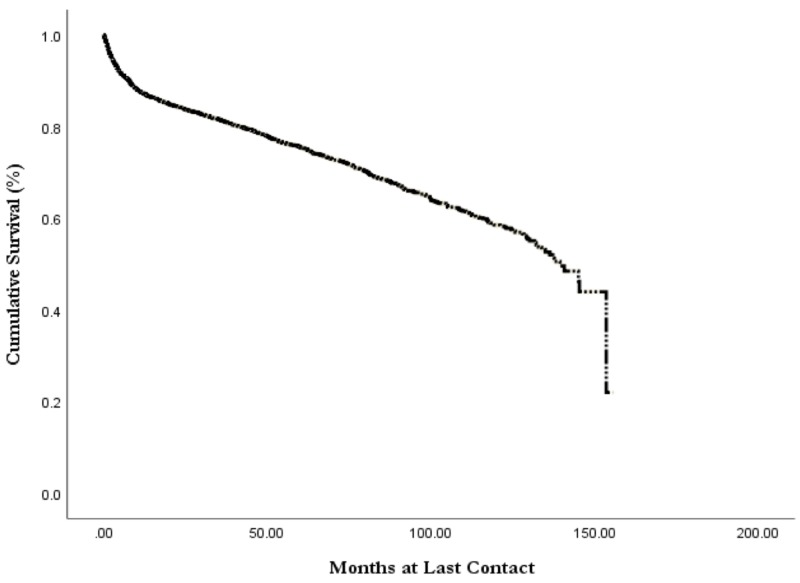
Overall all-cause survival of primary thyroid lymphoma from the NCDB, 2004-2015 NCDB, National Cancer Database

**Figure 2:**
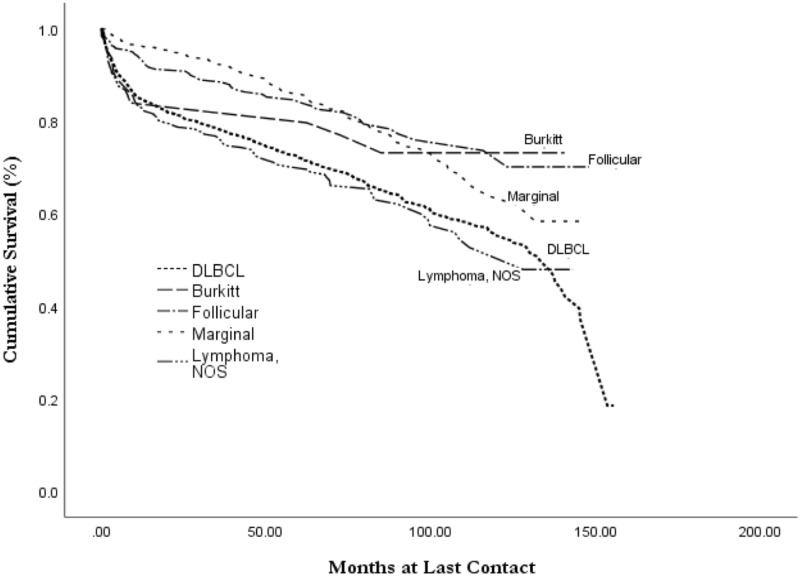
Overall survival for primary thyroid lymphoma by histology subtype, from the NCDB, 2004-2015 NCDB, National Cancer Database

**Figure 3:**
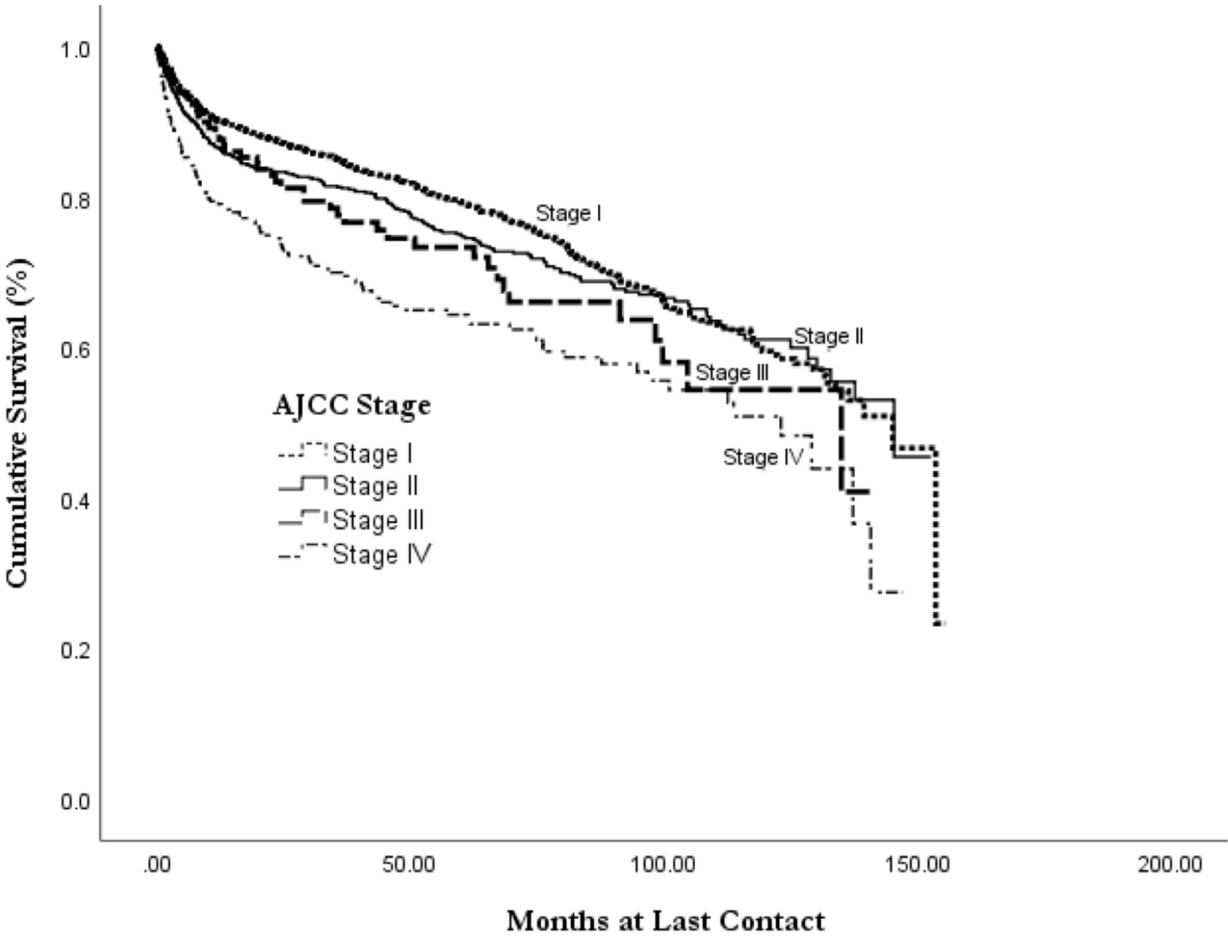
Overall survival of primary thyroid lymphoma by AJCC stage at diagnosis, from the NCDB 2004-2015 AJCC: American Joint Committee of Cancer; NCDB, National Cancer Database

Kaplan-Meier analysis revealed an overall all-cause median survival of 11.6 years (mean 9.0 years) with a 75% five-year and 59% 10-year survival (Table 4, Figure 1). Survival analysis by histology revealed that follicular lymphoma had the best mean survival at 10.2 years (*p* < 0.001; Figure 2). The worst survival of examined histologies was DLBCL, with a mean survival of 8.5 years. Burkitt’s lymphoma had a mean survival of 9.2 years (*p *> 0.05), and marginal lymphoma had a mean survival of 9.7 years (*p *< 0.001). Increased tumor stage was associated with decreased length of survival, with stage I survival of 9.3 years, stage II of 9.0 years, stage III of 8.1 years, and stage 4 having a survival of 7.5 years (Figure 3). 

**Table 4:** Mean, median, and cumulative five- and 10-year survival for primary thyroid lymphoma, with mean and cumulative five- and 10-year survival by histology and stage (from the NCDB, 2004-2015) * Reference; NS, not statistically significant (*p* > 0.05); ** not applicable; NCDB, National Cancer Database

Overall Survival				Cumulative Survival		
		Years	P value	5 year	10 year	P value
Total ( n= 3172)						
	(Mean)	9	**	75%	59%	**
	(Median)	11.6	**			
Mean Survival		Years	P value			
Histology (n = 3097)						
	DLBCL	8.5	*	70%	56%	*
	Burkitt	9.2	NS	79%	68%	NS
	Follicular	10.2	<0.001	84%	73%	<0.001
	Marginal	9.7	<0.001	86%	67%	<0.001
	Lymphoma, NOS	7.9	NS	68%	53%	NS
Stage (n = 2877)						
	I	9.3	*	78%	61%	*
	II	9	NS	73%	62%	<0.05
	III	8.1	NS	71%	55%	NS
	IV	7.5	<0.001	62%	50%	<0.001

The mean overall survival was examined for select treatment options (Table 5). Patients who did not receive surgical resection had a survival of 8.0 years. For those treated with surgery, lobectomy had the best survival at 9.9 years, with 9.7-year survival for total and subtotal thyroidectomy. Partial lobectomy and local excision had a survival of 8.4 years. Beam radiation had a significant (*p *< 0.001) improvement in survival over no radiation, with survival of 9.8 years compared to 8.3 years. Multiagent chemotherapy has a significant (*p *< 0.001) survival of 9.2 years over no chemotherapy, at 8.2 years.

**Table 5:** Mean overall survival by treatment method for primary thyroid lymphoma, from the NCDB, 2004-2015 * Reference; NS, not statistically significant (*p* >0.05); NCDB: National Cancer Database

Mean Survival		Years	P value
Surgical Treatment (n = 3423)			
	None	8.0	*
	Partial lobectomy/ local excision	8.4	NS
	Lobectomy	9.9	<0.001
	Total/ Subtotal Thyroidectomy	9.7	<0.001
Radiation Therapy (n = 3421)			
	None	8.3	*
	Beam Radiation	9.8	<0.001
	Radioisotopes	7.3	NS
Chemotherapy (n = 3263)			
	None	8.2	*
	Single-agent Chemotherapy	7.7	NS
	Multiagent Chemotherapy	9.2	<0.001

## Discussion

PTL is a rare thyroid neoplasm, representing only 1% to 5% of thyroid malignancies and 2.5% to 7% of all extranodal lymphomas [3,6,12]. To our knowledge, this represents the largest study to date examining the characteristics and survival of PTL, and the first study of the NCDB database. By analyzing this nationwide database to determine survival outcomes by demographics, tumor characteristics, and management strategies, opportunities for improvement in patient care may be identified. 

Demographics of the affected patients are consistent with previous studies, showing that a majority of patients are females, white, and non-Hispanic, with an average age of 65.8 years old [3-4,6]. The study of the SEER database by Graff-Baker, et al. supports these findings, where 75% of PTL patients were found to be female and 93% white, with an average age of 66.4 years [3]. To our knowledge, no prior study has examined the insurance status or facility type where patients received treatment. Our study documents the predominance of patients receiving care for this rare cancer at centers with higher caseloads and research, receiving treatment at academic (36.6%) and comprehensive community centers (43.9%) over community centers (9%). Most patients use private or Medicare insurance to cover treatment. The significant female predominance of PTL is especially notable when compared to lymphoma overall, as lymphoma is generally more common in males than females in almost all other subtypes [13]. 

Hashimoto’s thyroiditis is an established risk factor for PTL and a potential precursor, which may explain the female predominance and increased age range of patients when compared to other forms of lymphoma [2,5,13]. Hashimoto’s thyroiditis confers a 67 to 80-fold increase in the risk of development of PTL and has up to a 1:20 male to female ratio [2,9,12]. Hashimoto’s thyroiditis often accompanies PTL, with one case series of 38 patients finding pathological evidence of Hashimoto’s thyroiditis in up to 86% of patients with PTL [2]. Hashimoto’s thyroiditis can also be difficult to distinguish from PTL on fine needle aspiration [2,5]. PTL should be considered in patients with findings of thyroiditis on FNA that present with a rapidly enlarging neck mass and other signs not characteristics of Hashimoto's thyroiditis alone [2,5]. 

This study shows survival for PTL of median 11.6 years with a five-year overall survival of 75%, which is an improvement on prior studies. The largest previous study to date by Graff-Baker, et al. including 1,408 patients in the SEER database found a median all-cause survival of 9.3 years with a five-year overall survival of 66% [3]. The NCDB database used in this study includes patients diagnosed between 2004-2015, where Graff-Baker, et al. examined patients diagnosed between 1973-2005 [3]. Survival improvement in the current study may demonstrate improvement in diagnostic and treatment methods in recent years. Other estimates of survival have varied widely, with studies limited by sample size and follow-up. Derringer, et al. reported a 79% five-year survival, and other values from smaller studies range from 35% to 79% five-year disease-specific survival [3,6,7,14]. 

Multiple prior studies agree that DLBCL is the most common subtype of PTL, with other common histologies including marginal zone, or MALT lymphoma, and follicular lymphoma [3,4,6-8]. Cases of multiple other histologies have been reported, though these other histologies are rare and their incidence is not well defined [4,7]. Prior studies have found MALT lymphoma to be the second most common histology, with Graff-Baker, et al.finding 10% of all PTL patients in the SEER database presenting with MALT lymphoma [3,7]. This was supported by our study, finding 18.7% of cases were marginal zone or MALT lymphoma. In contrast, in a review of 108 cases of PTL, Derringer, et al. found a majority of PTL patients presenting with some form of MALT histology, whether as pure MALT or mixed MALT and DLBCL histology [6]. The histological classification of PTL is complicated by the potential for MALT lymphoma to transform into DLBCL, which may explain the variability between many case series and database studies [6,8]. 

This study also agrees with prior studies showing that DLBCL has the worst prognosis, with marginal (MALT) and follicular lymphoma having the best survival prognosis among common histologies [3,6,7,15]. Graff-Baker, et al. found a five-year disease-specific survival of 75% for DLBCL, 87% for follicular, 96% for MALT lymphoma, and 83% for other Non-Hodgkin’s lymphoma [3]. Though our analysis used all-cause mortality, we found similarly improved survival in follicular and marginal over DLBCL. Prior studies have noted that DLBCL generally presents at a more advanced stage, potentially leading to its poor outcomes [6]. 

Consistent with prior studies, most patients in the NCDB are diagnosed early at stage I, and this stage had a significant impact on overall survival. Graff-Baker, et al. found a five-year disease-specific survival of 86% for stage I, 81% for stage II, and 64% for stage III/IV [3]. Our analysis was limited by the lack of cancer-specific mortality in the NCDB database, finding lower five-year survival than Graff-Baker, et al., consistent with the use of all-cause mortality instead of cancer-specific survival [3]. However, the trend of decreased survival by increased stage is comparable.

PTL generally presents with a painless rapidly enlarging anterior neck mass that may cause obstructive symptoms and be associated with cervical lymphadenopathy [2-5,16]. Up to 10% of previously studied patients present with B type symptoms such as weight loss and night sweats and fever [2]. Our analysis confirms this finding, with 11.8% of patients presenting with systemic B symptoms at diagnosis. Bivariate analysis confirmed the findings by Graff-Baker, et al, that systemic symptoms increased the risk of mortality [3].

Optimal treatment methods have not been established and may vary by histology [16]. Treatment for PTL has generally been based on established treatment regimens for other extra-nodal non-Hodgkin’s lymphomas [2-4]. This treatment often varies by histology and stage, with a combination of multi-agent chemotherapy and immunotherapy generally used [2-4,7]. Loco-regional radiotherapy may be added depending on the size and spread of tumor [2-4,7]. Surgery once played a major role in the treatment of PTL, especially prior to the introduction of FNA biopsy in the 1970s [9]. The role of surgery has decreased as surgical biopsy and resection is no longer required for diagnosis, though surgery may still play a limited role in large tumors presenting with compressive symptoms [2]. However, many PTL tumors are still diagnosed from surgical specimen prior to histopathologic diagnosis, when surgery is performed for suspected thyroid cancer or palliation of compressive symptoms [2,9]. 

The majority (53.7%) of patients in the NCDB received some form of surgical resection [2-3,5,9]. Graff-Baker, et al. found comparatively high rates of surgical treatment in the SEER database, at 61% [3]. This is higher than expected for a malignancy where the recommended treatment is chemotherapy with or without radiation [3,4]. These non-invasive treatment options have a significant mortality benefit, and some studies have even gone as far as to suggest that surgery has no role in the therapeutic management of PTL beyond diagnosis [5,14]. Graff-Baker, et al. hypothesized that the high surgical volume may be due to debulking in advanced disease, the desire for definitive treatment in stage I disease, inappropriate surgeries, or the diagnosis of PTL only after resection for a separate thyroid pathology [3]. This large incidence of surgical management may also be due to a holdover of historical treatment methods, where surgical debulking was once the primary method of management [5,9]. Though not generally recommended as first-line therapy, surgery had significant survival benefit on multivariate analysis. Even with controlling for stage and histology, lobectomy and total or subtotal thyroidectomy showed a significant survival benefit.

Chemotherapy, which is generally the recommended treatment, usually involves the CHOP regimen of cyclophosphamide, doxorubicin (hydroxydoxorubicin), vincristine (Oncovin), and prednisone [2,9]. Additionally, the immunotherapy agent rituximab is often added to this combination, with CHOP plus rituximab showing promising results in the management of PTL, though studies that specifically address PTL remain small [2]. 

As the first population study examining chemotherapy, we found that only 31.7% of patients did not receive chemotherapy. The majority (61.5%) received multi-agent treatment, though information on specific chemotherapeutic regimens is not available in the NCDB. Multi-agent and single-agent chemotherapy provide a significant improvement in survival, even when controlled for stage, histology, and other factors. Bivariate analysis also showed a significant survival benefit for neoadjuvant chemotherapy following surgical resection. This data supports the use of chemotherapy as the primary treatment regimen for PTL.

Specific regimens are not available in the NCDB, and all immunotherapies, including Rituximab, are placed in one category. Only 16.2% of patients reportedly received immunotherapy, which may be due to its more recent introduction into the market, though it is most likely due to inconsistency in the definition and coding of immunotherapy into the NCDB database. Our survival analysis showed a benefit to its use, though these findings were not statistically significant. Further studies will be needed to support the benefit of immunotherapy, especially Rituximab, in PTL [2]. 

Prior studies have found that radiation provides significant benefit to patients with PTL, though only 41.6% of our patients received radiation treatment [2-3,6]. Beam radiation had significant survival benefit to patients with PTL on multivariate analysis, with a survival benefit of 1.5 years over patients who did not receive radiation. Adjuvant radiation following surgical resection was also significantly beneficial, though only 22.4% of patients received this sequence of treatment. Graff-Baker, et al. found similar survival benefit to surgery and radiation [3]. With the large number of patients receiving surgical management for PTL, there may be a greater role for adjuvant radiation than is currently observed. 

## Conclusions

This study represents the largest analysis of PTL patients to date and is the first population study to examine the use of chemotherapy. PTL remains rare, that is, heterogeneous in its histology and presentation. The majority of PTL is non-Hodgkin’s lymphoma of B-cell origin, with DLBCL representing the most common histology, followed by marginal zone and follicular lymphoma. The most common treatment regimen is multiagent chemotherapy, which has the best survival of all treatment modalities. Surgical resection and radiation therapy are also commonly used and both show a significant survival benefit. With current treatment options, the overall prognosis of PTL is good, with median all-cause survival of 11.6 years, and a five- and 10-year survival of 75% and 59%. Increased risk of death is associated with increasing age, DLBCL histology, and higher disease stage. Improved outcomes are associated with treatment at academic and integrated centers and with marginal and follicular histology.

Further research is needed to establish optimum treatment guidelines, especially within histological subtypes and by stage. Continued research is particularly important in comparing treatment modalities of surgery, chemotherapy, and radiation, especially with the introduction of new immunotherapies, such as Rituximab, and in comparing surgical approaches.
